# Spatiotemporal Kernel Reconstruction for Linear Parametric Neurotransmitter PET Kinetic Modeling in Motion Correction Brain PET of Awake Rats

**DOI:** 10.3389/fnins.2022.901091

**Published:** 2022-05-12

**Authors:** Alan Miranda, Daniele Bertoglio, Sigrid Stroobants, Steven Staelens, Jeroen Verhaeghe

**Affiliations:** ^1^Molecular Imaging Center Antwerp, University of Antwerp, Antwerp, Belgium; ^2^Department of Nuclear Medicine, University Hospital Antwerp, Antwerp, Belgium

**Keywords:** positron emission tomography, brain, rat, motion correction, 4D reconstruction, kinetic modeling

## Abstract

The linear parametric neurotransmitter positron emission tomography (lp-ntPET) kinetic model can be used to detect transient changes (activation) in endogenous neurotransmitter levels. Preclinical PET scans in awake animals can be performed to investigate neurotransmitter transient changes. Here we use the spatiotemporal kernel reconstruction (Kernel) for noise reduction in dynamic PET, and lp-ntPET kinetic modeling. Kernel is adapted for motion correction reconstruction, applied in awake rat PET scans. We performed 2D rat brain phantom simulation using the ntPET model at 3 different noise levels. Data was reconstructed with independent frame reconstruction (IFR), IFR with HYPR denoising, and Kernel, and lp-ntPET kinetic parameters (*k*_*2a*_: efflux rate, γ: activation magnitude, *t*_*d*_: activation onset time, and *t*_*p*_: activation peak time) were calculated. Additionally, significant activation magnitude (γ) difference with respect to a region with no activation (rest) was calculated. Finally, [^11^C]raclopride experiments were performed in anesthetized and awake rats, injecting cold raclopride at 20 min after scan start to simulate endogenous neurotransmitter release. For simulated data at the regional level, IFR coefficient of variation (COV) of *k*_*2a*_, γ, *t*_*d*_ and *t*_*p*_ was reduced with HYPR denoising, but Kernel showed the lowest COV (2 fold reduction compared with IFR). At the pixel level the same trend is observed for *k*_*2a*_, γ, *t*_*d*_ and *t*_*p*_ COV, but reduction is larger with Kernel compared with IFR (10–14 fold). Bias in γ with respect with noise-free values was additionally reduced using Kernel (difference of 292, 72.4, and −6.92% for IFR, IFR+KYPR, and Kernel, respectively). Significant difference in activation between the rest and active region could be detected at a simulated activation of 160% for IFR and IFR+HYPR, and of 120% for Kernel. In rat experiments, lp-ntPET parameters have better confidence intervals using Kernel. In the γ, and *t*_*d*_ parametric maps, the striatum structure can be identified with Kernel but not with IFR. Striatum voxel-wise γ, *t*_*d*_ and *t*_*p*_ values have lower variability using Kernel compared with IFR and IFR+HYPR. The spatiotemporal kernel reconstruction adapted for motion correction reconstruction allows to improve lp-ntPET kinetic modeling noise in awake rat studies, as well as detection of subtle neurotransmitter activations.

## Introduction

Transient changes in brain neurotransmitter levels can be investigated with dynamic positron emission tomography (PET) using for example the linear parametric neurotransmitter PET kinetic model (lp-ntPET) (Morris et al., 2005; Normandin et al., 2012). Transient changes in dopamine levels due to a rewarded task (Pappata et al., 2002), a motor planning task (Alpert et al., 2003), and due to psychosocial stress (Lataster et al., 2011), have been investigated in human brain PET. More recently, the effect of smoking (Cosgrove et al., 2014), gambling (Bevington et al., 2021), and cannabis (Calakos et al., 2021) on transient dopamine release has been investigated with the lp-ntPET method. These type of studies are not possible to perform in typical preclinical PET scans in which the animal is anesthetized. Therefore, the use of motion correction techniques, allowing to scan animals in the awake state (Kyme et al., 2014; Spangler-Bickell et al., 2016; Miranda et al., 2017), would make possible to investigate transient changes in neurotransmitter levels caused by a task or external stimuli in preclinical PET. Methods that involve head motion tracking followed by motion correction have been developed and improved over the last years to perform scans in awake rodents (Kyme et al., 2014; Spangler-Bickell et al., 2016; Miranda et al., 2017).

Using the lp-ntPET model (Normandin et al., 2012), the transient activation of certain neurotransmitter receptors can be quantified using tracers targeting these receptors (e.g., [^11^C]raclopride for dopamine D_2/3_ receptors) (Kyme et al., 2019). By modeling the tracer efflux in compartment modeling as a time varying parameter (Normandin et al., 2012), transient changes in endogenous neurotransmitter concentrations can be inferred by transient changes in tracer binding. For instance, the lp-ntPET has been used to quantify the striatal transient dopamine activation profile in awake rats following an amphetamine challenge (Kyme et al., 2019).

In order to perform kinetic modeling, dynamic PET reconstruction is necessary to determine the tracer concentration over time. Independent reconstruction of every time frame is the straightforward method to perform dynamic PET, but frame images, and therefore kinetic parameters, usually have high noise level due to the small number of events in each frame. To reduce noise in dynamic PET and kinetic modeling parameters, a wide variety of methods can be implemented (Reader and Verhaeghe, 2014; Wang et al., 2020), such as post-processing using the highly constrained backprojection method (Christian et al., 2010), or using machine learning denoising (Reader et al., 2021). Particularly for the case of dynamic PET for kinetic modeling, direct reconstruction has been developed to reduce noise (Matthews et al., 2010). In this method, the kinetic model is fitted to every voxel after every reconstruction iteration and therefore parametric images can be calculated during reconstruction. This method has been applied using the lp-ntPET kinetic model for noise reduction (Angelis et al., 2019). Another reconstruction developed for noise reduction in dynamic PET is the kernel method (Wang and Qi, 2015; Wang, 2019; Miranda et al., 2021). This method makes use of spatial and temporal correlations in the data to reduce noise in the iterative reconstruction (Wang and Qi, 2015; Novosad and Reader, 2016; Wang, 2019).

In this work, the lp-ntPET kinetic model was used to quantify transient dopamine changes in the rat striatum. As a first objective, we validated the spatiotemporal kernel reconstruction for lp-ntPET kinetic modeling in a 2D simulation and compare it with independent frame reconstruction. Then, we adapted the spatiotemporal kernel reconstruction for motion correction reconstruction to enable it in awake small animal scans. The method was used to perform a [^11^C]raclopride scan, using cold raclopride as challenge, in an awake freely-moving rat using the point source tracking method (Miranda et al., 2017).

## Materials and Methods

### Motion Tracking and Independent Frame Motion Correction Reconstruction

The rat head motion in awake rat scans was tracked using the point source tracking method (Miranda et al., 2017). Four point sources prepared with [^18^F]FDG were attached on the rat head. Two point sources were attached below each ear, one on the nose bridge, and one in between the right ear and nose. Each point source was prepared with [^18^F]FDG and had an activity in the range of 222-370 kBq.

Animals were scanned on an Inveon PET scanner (Siemens Medical Solutions, Inc., Knoxville, United States). Images are reconstructed in a grid of 128 × 128 × 159 voxels with a size of 0.776 × 0.776 × 0.796 mm along the *x*, *y* and *z* directions, respectively. Independent frame motion correction reconstruction was calculated using list-mode event-by-event motion correction (LMMC) with 16 subsets and 8 iterations (Rahmim et al., 2008). The sensitivity image for motion correction was calculated by interpolation in the image space (Rahmim et al., 2008). The attenuation map was calculated using the binary image of the activity body outline with an uniform attenuation factor for soft tissue (0.096 cm^–1^) (Angelis et al., 2013). Motion dependent and spatially variant resolution modeling was implemented as well (Miranda et al., 2020). Dynamic images were reconstructed with a framing of 12 frames × 10 s, 6 ×20 s, 2 ×60 s, and 27 ×120 s.

### Spatiotemporal Kernel Reconstruction for Motion Correction

The spatiotemporal kernel method (Wang and Qi, 2015; Wang, 2019) has been adapted for the case of PET rigid motion correction reconstruction. Briefly, the original method to calculate the spatial kernel matrix consists of dividing the entire PET scan in 3 frames and use the voxel intensity values of these 3 frames as the feature of the corresponding voxel. Using a Gaussian radial kernel, the correlation between voxels is calculated, which serve as the values of the spatial kernel matrix elements. For the case of small animal brain motion correction, the region of the image outside the head of the animal is not corrected for motion, and therefore it can be affected by blurring motion. Therefore, we define a rectangular region enclosing the animal head to calculate the spatial correlations for voxels only in that region. We use a neighborhood of 9 ×9 ×9 voxels, and a threshold of 0.8 in the radial Gaussian kernel value, to calculate the spatial kernel matrix. Only the 48 closest nearest neighbors were considered to create the sparse spatial kernel matrix.

To calculate the temporal kernel matrix, originally it was proposed to use the sinogram as the frame feature to calculate the correlation between frames (Wang, 2019). Although is possible to perform sinogram rebinning to calculate the motion corrected sinogram (Rahmim et al., 2008), these sinograms often present gaps due to the position of the detectors after motion correction which do not overlap the sinogram space. These gaps can differ between frames, and the effect can be pronounced in small animal brain scans, in which the animal head can have a wide range of orientations. For this reason, we replaced the sinogram with an approximate LMMC reconstruction as the frame feature. LMMC reconstruction allows to consider all events after motion correction for reconstruction (i.e., no events are discarded due to falling out of the sinogram space), and the motion corrected sensitivity image corrects for non-uniformities due to motion compensation (Rahmim et al., 2008). We calculate the approximate LMMC of every frame considering 16 subsets and only one iteration, without attenuation correction or resolution modeling. This smooth reconstruction also allows to reduce the difference between voxel kinetics, and therefore improve correlation between frames to produce temporal basis functions that can model the different kinetics present in the image. The point sources are masked from every frame image before calculating the frames features correlation with the radial Gaussian kernel (Wang, 2019). To reduce noise in the temporal basis functions calculated from the correlation between frames, we filter every temporal basis function using a Gaussian filter with σ = ts/100, were ts = 15 is the size of the frame neighborhood to calculate the correlation with other frames.

### Highly Constrained Backprojection Denoising

Since the highly constrained backprojection (HYPR) denoising (Christian et al., 2010) has been shown to improve parameter estimation in lp-ntPET kinetic modeling (Wang et al., 2017), we applied HYPR denoising to independent frame reconstruction dynamic images. The time averaged sum of dynamic frames was used as the composite image, and a 3 ×3 ×3 boxcar filter was used to perform HYPR filtering in dynamic frames (Christian et al., 2010).

### 2D ntPET Simulation

**FIGURE 1:**
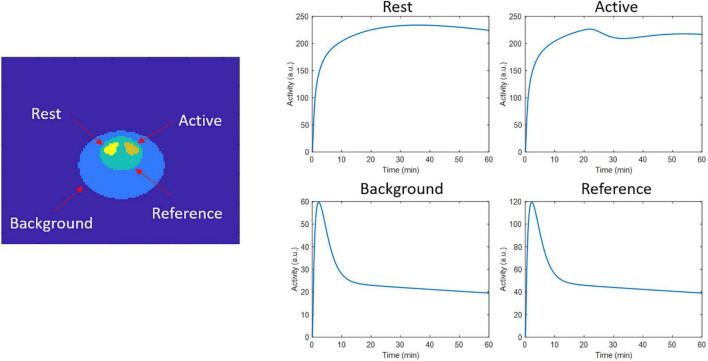
2D phantom regions and time activity curves used to generate the dynamic events data in the respective regions.

In order to validate the spatiotemporal reconstruction for kinetic modeling using lp-ntPET, we performed a 2D simulation of a brain phantom using the ntPET model (Morris et al., 2005; Normandin and Morris, 2008). Similar to Angelis et al. (2019), we simulated a rat brain phantom with the striatum structure, where the left striatum did not present endogenous neurotransmitter activation (rest region), while the right striatum was activated (active region). A reference region, necessary to perform lp-ntPET kinetic modeling, was considered as well. The time activity curves (TACs) of the reference, rest, and active regions were generated for a 60 min scan with the same parameters as in Angelis et al. (2019). These parameters consider an activation profile peak of 200% the basal dopamine level (reducing dopamine binding by 10%), an onset activation time of 20 min, with a peak time at 25 min. Figure 1 shows the phantom image, and the TACs from the different regions. The simulation was performed considering [^11^C] raclopride to simulate tracer decay, and incorporating photon attenuation with a uniform attenuation factor for soft tissue for the entire head. The image had a size of 128 ×128 pixels with a pixel size of 0.776 × 0.776 mm. List-mode data frames were generated with the same framing used for dynamic image reconstruction (section motion tracking and independent frame motion correction reconstruction). Simulations with 10, 40, and 80 million counts were generated, considering 30 realizations per count level. Data was reconstructed with independent frame reconstruction and spatiotemporal kernel reconstruction with 300 iterations in both cases.

**FIGURE 2:**
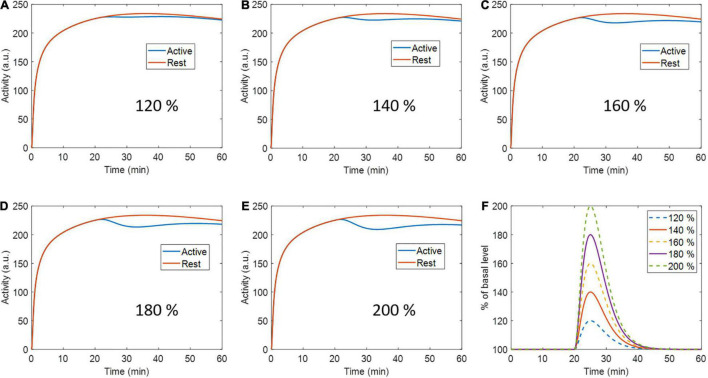
Active region (and rest region for reference) time activity curves with **(A)** 120 %, **(B)** 140 %, **(C)** 160%, **(D)** 180 %, and **(E)** 200 % percent peak dopamine release with respect to baseline. **(F)** Activation profiles for every respective level of activation.

A second set of simulations were performed with the same previously described phantom and ntPET model parameters, but at 5 different peak levels of activation: 120, 140, 160, 180, and 200% the basal level (Figure 2). Ten realizations were calculated per activation level, with 40 million counts in all cases.

### Awake Rat Cold Raclopride Brain Scans

**FIGURE 3:**
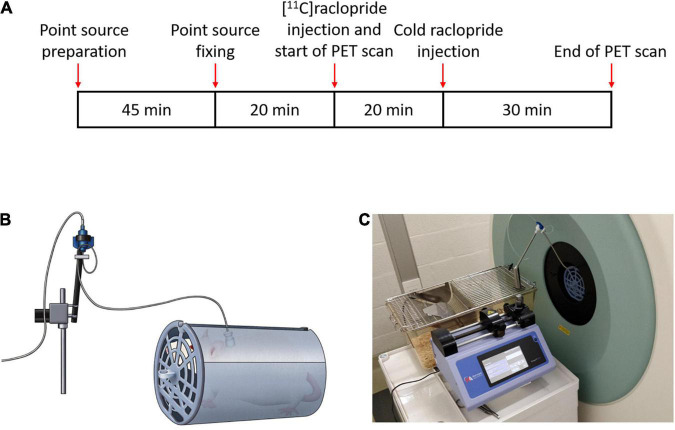
**(A)** Scanning time schedules for the awake cold raclopride scan. **(B)** Diagram of the rat inside the holder tube connected to the line swivel and **(C)** scanner setup showing the injection pump and swivel.

In order to perform the injection of the tracer in the awake state, a catheter was initially implanted in the jugular vein (Feng et al., 2015) in 2 Wistar female rats (Janvier Labs). Surgery was performed under isoflurane anesthesia (5% for induction, 1.5% for maintenance). After surgery, rats were left to rest during one week, followed by 3 days of acclimatization inside the holder used to maintain the rats inside the scanner field of view (Figure 3B). Catheter was flushed with heparin solution for maintenance every day for one week, and 2–3 times per week afterwards. The experiments followed the European Ethics Committee recommendations (decree 86/609/CEE) and were approved by the Animal Experimental Ethical Committee of the University of Antwerp, Antwerp, Belgium (ECD 2016-89).

Two rats underwent a cold raclopride challenge scan, one under anesthesia (210 g) and the other in the awake state (197 g). For the scans under anesthesia, the rat was initially administered with isoflurane (5% for induction, 1.5% for maintenance) and placed on the scanner bed. At the start of the 60 min PET scan, the rat was administered with [^11^C]raclopride (11.6 MBq, Molar activity, MA: 38.2 MBq/nmol) through the jugular vein catheter. Twenty minutes after the start of the scan, cold raclopride in 0.2 mL saline (1 mg/kg) was administered. This dose was chosen to observe a clear displacement of [^11^C]raclopride (Wadenberg et al., 2000; Kyme et al., 2019). For the awake scan (Figure 3A), 20 min before the start of the scan, four point sources were attached on the rat head in the awake state. At the onset of the PET scan, [^11^C]raclopride was administered through the jugular vein catheter (12.4 MBq, MA: 45.3 MBq/nmol). Twenty minutes after the start of the scan, cold raclopride (1 mg/kg) in 0.2 mL saline was administered through the jugular vein catheter.

### Kinetic Modeling

In both simulations and experimental data, the lp-ntPET kinetic model (Normandin et al., 2012) was used to calculate the magnitude and time of the activation profile. The lp-ntPET kinetic model represents the tissue TAC as a function of the reference region TAC as (Alpert et al., 2003; Normandin et al., 2012):


(1) $$C_{T}\,\left(t\right)=R_{1}\,C_{R}\,\left(t\right)+k_{2}\,\int_{0}^{t}C_{R}\,\left(u\right)\,du\;-k_{2\,a}\,\int_{0}^{t}C_{T}\,\left(u\right)\,du-\gamma \,B_{i}\,\left(t\right)$$

where *C_T_* is the activity in tissue, *C_R_* the activity in the reference tissue (cerebellum in our case), *R_1_* the ratio of the delivery in tissue compared to the reference tissue, *k_2_* is the rate constant transfer from free compartment to plasma, *k*_*2a*_ is the apparent rate constant transfer from specific compartment to plasma, and γ is the magnitude of the activation response modeled with basis functions *B*_*i*_(*t*):


(2) $$B_{i}\,\left(t\right)=\int_{0}^{t}C_{T}\,\left(u\right)\,h_{i}\,\left(u\right)\,du$$

where *h*_*i*_(*t*) is modeled with a gamma variate function:


(3) $$h_{i}\,\left(t\right)={\left(\frac{t-t_{d}}{t_{p}-t_{d}}\right)}^{\alpha }\,\text{exp}\,\left(\alpha \,\left[1-\frac{t-t_{d}}{t_{p}-t_{d}}\right]\right)\,u\,\left(t-t_{d}\right)$$

where *t*_*d*_ is the delay time (from injection onset) at which the activation starts, *t*_*p*_ is the peak time of the activation, α determines the skewness of the activation, and *u*(*t*) is the Heaviside function. Every *i*-th *h*_*i*_(*t*) function is calculated with a different combination of *t*_*d*_, *t*_*p*_ and α parameters, with the following ranges: *t*_*d*_ ranged from 10 to 40 min, in intervals of 1.5 min, *t*_*p*_ depended on *t*_*d*_ and ranged from *t*_*d*_ to *t*_*end*_− 5 min (*t*_*end*_:scan end time) in intervals of 1.5 min, and α ranged from 0.5 to 3, in intervals of 0.5. A total of 2,394 basis functions were calculated using (2) and (3). The activation response profile (ARP) is reported as the percentage of change in baseline dopamine efflux (*k*_*2a*_):


(4) $$\text{ARP}=100\times \frac{\gamma \,h_{i}\,\left(t\right)}{k_{2\,a}}$$

Since either in simulation experiments or in animal scans, a decrease in [^11^C] raclopride binding is expected, non-negative linear least-squares was used to calculate the set of parameters [*R*_1_*k*_2_*k*_2*a*_γ] with all basis functions and selecting the solution with the minimum least squared error from all basis functions. Parameters [*t*_*d*_*t*_*p*_α] are obtained from the basis function which results in the minimum least squared error. If no prior information about the decrease/increase of dopamine is known, linear least squares (i.e., allowing positive and negative γ magnitude) should be used.

### Data Analysis

From simulation data, the mean and standard deviation (SD) of the activation parameters of interest, i.e., *k*_*2a*_, γ, *t*_*d*_, and *t*_*p*_, over all realizations at the 3 different count levels, was calculated for independent frame reconstruction (IFR), spatiotemporal kernel reconstruction (Kernel), and IFR with HYPR denoising (IFR+HYPR). The relative difference with respect to the noise-free parameters is calculated as well. Mean and SD parametric γ, *t*_*d*_, and *t*_*p*_ maps are calculated for the different count levels.

For the simulation with different activation profile levels, the relative magnitude of γ, i.e., the ratio γ/*k*_2*a*_, which indicates the activation magnitude relative to the baseline washout, was calculated for the active and rest region. A paired *t*-test was calculated between rest and active regions γ/*k*_2*a*_ ratios at the 5 different activation levels for IFR, Kernel, and IFR+HYPR.

For the experimental data cold raclopride scans, a single frame reconstruction was used to manually align the rat brain to an MRI template with delineated striatum and cerebellum regions. TACs were extracted from these regions using PMOD 3.6 (Pmod technologies, Zurich, Switzerland). Kinetic modeling was performed and regional striatum *k*_*2a*_, γ, *t*_*d*_, and *t*_*p*_ parameters were calculated. In addition, the approximate Bayesian computation (ABC) framework (Toni et al., 2009; Kyme et al., 2019; Fan et al., 2021) was used to calculate the confidence intervals of the parameters *k*_*2a*_, γ, *t*_*d*_, and *t*_*p*_ in experimental data. For the prior, using the parameters obtained from the solution of least squares error, we considered a uniform distribution, with limits at +-100% the best fit values, considering 10 million sampling trials. The tolerance, obtained by trial and error as in Fan et al. (2021) was set at 2 times the least square error fit. Finally, *t*_*d*_, *t*_*p*_, and γ and its t-statistic (γ/SE(γ)) voxel-wise parametric brain maps, were calculated.

## Results

### 2D ntPET Simulation

**FIGURE 4:**
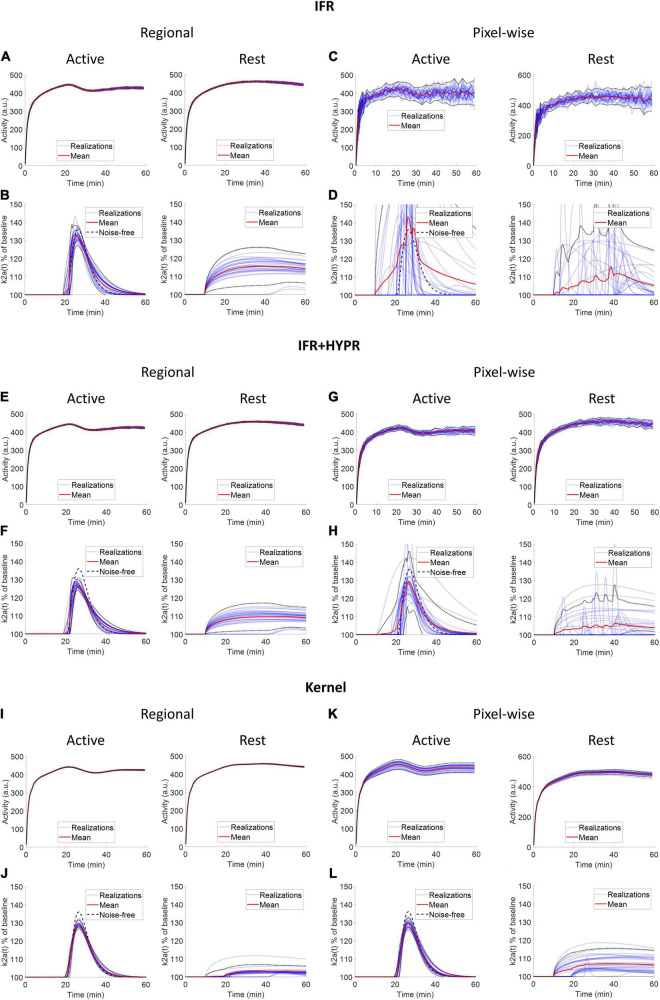
Individual and mean TACs for every realization, at the regional **(A,B,E,F,I,J)** and pixel level **(C,D,G,H,K,L)**, and activation response profiles (ARP) for the active and rest regions, using independent frame reconstruction (IFR), independent frame reconstruction with HYPR denoising (IFR+HYPR), and spatiotemporal kernel reconstruction (Kernel), for simulations with 80 million counts. Dotted lines show 2 SD.

Figure 4 shows the regional and a pixel-wise TACs from the active and rest regions, and the ARP from the respective TACs, for noise realizations with 80 million counts. For IFR at the regional level, TACs present little variation (mean coefficient of variation, COV, along the TAC: 1.20%), however, the ARP present larger variation (COV: 2.59%). The mean magnitude of the ARP is higher in the active region compared to the rest region and has a sharper profile. At the pixel level, TACs present high noise (COV: 10%), and ARP have large magnitude differences across realizations (COV: 22.8%). However, mean active ARP is higher than in the rest region, and peak time in active ARP coincides with the noise-free ARP. Denoising IFR with HYPR reduces variation in active region TACs and ARP, both at the regional (TAC: 0.94%, ARP: 1.72%) and pixel level (4.01%, 5.99%), with better resemblance in ARP shape compared to noise-free ARP, although with reduced magnitude. For the Kernel reconstruction at the regional level, both TAC and ARP variation is further reduced (0.40 and 0.90%, respectively) compared to IFR and IFR+HYPR. Moreover, magnitude of the rest ARP is reduced using Kernel compared to IFR. For Kernel at the pixel level, variation in the active TAC (2.2%) is similar to IFR variation at the regional level (1.19%). Noise in ARP is greatly reduced at the pixel level using Kernel (1.46%) compared to IFR (22.8%), and IFR+HYPR (5.99%), with lower variation at the regional level than IFR (2.59%) and IFR+HYPR (1.72%). Corresponding plots for 40 and 10 million counts simulations are shown in Supplementary Figures 1, 2, respectively. Noise increases with lower counts for all methods. At the pixel level for 10 million counts, the shape of the ARP is greatly distorted for IFR and improved in IFR+HYPR, but the mean peak time (32 min) still is close to the noise-free value. On the other hand, Kernel ARP are less distorted and with good peak time (26 min) correspondence with the noise-free ARP.

Table 1 show the lp-ntPET kinetic modeling parameters of interest for the simulations with all 3 count levels, for IFR, IFR+HYPR and Kernel. At the regional level, coefficient of variation is improved in IFR when HYPR denoising is used, with Kernel further reducing variability. On the other hand, relative difference of *k*_*2a*_ and γ with respect to the noise-free value is larger using IFR+HYPR and Kernel compared with IFR, but *t*_*d*_ and *t*_*p*_ relative difference is lower using Kernel compared to IFR and IFR+HYPR.

**TABLE 1:** Regional lp-ntPET kinetic modeling *k*_*2a*_, γ, *t*_*d*_ and *t*_*p*_ mean, coefficient of variation (COV), and difference with respect to noise-free value, for independent frame reconstruction (IFR), independent frame reconstruction with HYPR denoising (IFR+HYPR), and spatiotemporal kernel reconstructions (Kernel), in simulations with 80, 40 and 10 million counts.

	Noise-free	IFR			IFR+HYPR			Kernel		
		Mean	COV	Difference	Mean	COV	Difference	Mean	COV	Difference
		80 million								
*k*_*2a*_ (min^–1^)	0.0522	0.0557	2.80%	6.65%	0.0632	2.44%	20.89%	0.0576	1.28%	10.2%
γ (min^–1^)	0.0187	0.0185	10.5%	^–^1.27%	0.0173	5.87%	^–^7.58%	0.0167	4.13%	^–^10.7%
*t*_*d*_(min)	20.5	21.2	6.02%	3.54%	21.4	4.72%	4.25%	20.4	3.25%	^–^0.47%
*t*_*p*_(min)	26.5	25.6	2.94%	^–^3.29%	25.6	2.94%	^–^3.29%	26.5	0.00%	0.00%
		40 million								
*k*_*2a*_ (min^–1^)	0.0522	0.0556	3.69%	6.53%	0.0630	3.39%	20.59%	0.0576	2.06%	10.2%
γ (min^–1^)	0.0187	0.0183	13.7%	^–^2.42%	0.0169	8.47%	^–^9.91%	0.0170	4.29%	^–^9.48%
*t*_*d*_(min)	20.5	21	8.25%	2.44%	20.8	8.12%	1.46%	20.4	5.61%	^–^0.24%
*t*_*p*_(min)	26.5	25.7	3.33%	^–^3.02%	25.7	3.33%	^–^2.83%	26.5	0.00%	0.00%
		10 million								
*k*_*2a*_ (min^–1^)	0.0522	0.0551	7.10%	5.62%	0.0624	5.74%	19.46%	0.0573	2.88%	9.69%
γ (min^–1^)	0.0187	0.0204	26.5%	8.69%	0.0177	20.17%	^–^5.79%	0.0168	9.19%	^–^10.2%
*t*_*d*_(min)	20.5	21.0	10.8%	2.68%	20.8	8.80%	1.71%	20.5	6.50%	0.24%
*t*_*p*_(min)	26.5	25.8	3.97%	^–^2.83%	25.6	4.51%	^–^3.40%	26.2	2.77%	^–^1.13%

Similar to Tables 1, 2 shows kinetic modeling statistics, but at the pixel level. As in the regional analysis, at the pixel level for all count levels, coefficient of variation is larger for IFR, with HYPR denoising reducing variability. Kernel shows the lowest COV for all parameters at all noise levels. Except for *k*_*2a*_ at 80 and 40 million counts, Kernel shows smaller difference with respect to noise-free values than IFR. Particularly, γ present large differences with respect to noise-free values using IFR.

**TABLE 2:** Pixel-wise lp-ntPET kinetic modeling *k*_*2a*_, γ, *t*_*d*_ and *t*_*p*_ mean, coefficient of variation (COV), and difference with respect to noise-free value, for independent frame reconstruction (IFR), independent frame reconstruction with HYPR denoising (IFR+HYPR), and spatiotemporal kernel reconstructions (Kernel), in simulations with 80, 40 and 10 million counts.

	Noise-free	IFR			IFR+HYPR			Kernel		
		Mean	COV	Difference	Mean	COV	Difference	Mean	COV	Difference
		80 million								
*k*_*2a*_ (min^–1^)	0.0522	0.0558	32.7%	6.83%	0.0642	6.96%	22.86%	0.0585	1.76%	12.1%
γ (min^–1^)	0.0187	0.0265	126%	41.2%	0.0191	30.30%	1.98%	0.0172	5.30%	^–^8.39%
*t*_*d*_(min)	20.5	23.2	37.7%	13.4%	20.5	14.01%	0.00%	20.4	4.22%	^–^0.47%
*t*_*p*_(min)	26.5	28.7	25.9%	8.22%	25.7	3.65%	^–^2.92%	26.5	0.00%	0.00%
		40 million								
*k*_*2a*_ (min^–1^)	0.0522	0.0493	32.2%	^–^5.55%	0.0626	15.5%	19.79%	0.0587	2.71%	12.3%
γ (min^–1^)	0.0187	0.0482	50.3%	156%	0.0229	35.8%	21.86%	0.0174	7.20%	^–^7.21%
*t*_*d*_(min)	20.5	22.9	30.0%	11.9%	20.1	23.1%	^–^1.95%	20.4	6.24%	^–^0.24%
*t*_*p*_(min)	26.5	28.6	26.8%	7.92%	25.9	4.4%	^–^2.08%	26.5	0.00%	0.00%
		10 million								
*k*_*2a*_ (min^–1^)	0.0522	0.0396	54.7%	^–^24.0%	0.0590	21.2%	12.94%	0.0569	6.30%	8.88%
γ (min^–1^)	0.0187	0.0736	74.8%	292%	0.0324	45.8%	72.43%	0.0175	15.8%	^–^6.92%
*t*_*d*_(min)	20.5	24.9	40.8%	21.7%	21.1	30.8%	3.17%	20.1	9.10%	^–^1.71%
*t*_*p*_(min)	26.5	32.25	31.5%	21.7%	26.6	15.8%	0.38%	26.3	3.12%	^–^0.57%

**FIGURE 5:**
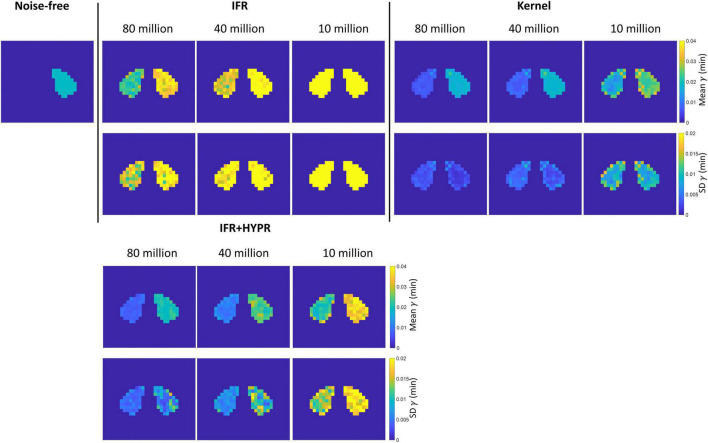
Rest and active parametric γ mean **(top row)** and standard deviation **(bottom row)** images, for independent frame reconstruction (IFR), independent frame reconstruction with HYPR denoising (IFR+HYPR), and spatiotemporal kernel reconstructions (Kernel).

Figure 5 shows the mean and SD γ parametric maps for the different count levels using IFR and Kernel. For the rest region, as also shown in Table 2, magnitude of γ becomes larger using IFR and IFR+HYPR at increasing noise levels, while using Kernel, magnitude is similar across noise levels. Standard deviation is larger in the active region compared to the rest region pixels using IFR and IFR+HYPR, but lower using Kernel. For all count levels and both regions, standard deviation is lower using Kernel. Supplementary Figures 3, 4 show the mean and SD *t*_*d*_ and *t*_*p*_ parametric maps for the different count levels using IFR, IFR+HYPR, and Kernel. As with γ, *t*_*d*_ Kernel parametric maps are similar across noise levels and with lower standard deviation compared with IFR and IFR+HYPR in the active region, but IFR+HYPR reduce noise to a level similar to that in the Kernel reconstruction. On the other hand *t*_*p*_ parametric maps are similar using IFR, IFR+HYPR and Kernel, with Kernel presenting lower standard deviation in 80 and 40 million counts, but IFR+HYPR showing lower SD in 10 million counts in the active region. Indeed, *t*_*p*_ was the parameter with lowest COV for IFR and IFR+HYPR as shown in Table 2.

Table 3 shows the relative magnitude of γ (γ/*k*_2*a*_) for different peak activation levels, and the difference between active and rest region relative magnitude. Using IFR, no significant difference in γ/*k*_2*a*_ was found for peak activation levels of 120 and 140%, but with HYPR denoising difference at 120% becomes significant. Significant difference in IFR is found for 160, 180, and 200% peak activation levels, with highest significance in the 200% level. For Kernel, difference between active and rest γ/*k*_2*a*_ is significant for all activation peak levels (p**< 0.01), reaching a significance of p****< 0.0001 for the activation levels higher or equal to 140%. In addition, γ/*k*_2*a*_ increase in proportion to the activation peak level (120%: 0.088, 140%: 0.142, 160%: 0.199, 180%: 0.250, 200%: 0.295) using Kernel, while this is not observed using IFR (120%: 0.263, 140%: 0.159, 160%: 0.223, 180%: 0.282, 200%: 0.330) or IFR+HYPR.

**TABLE 3:** Mean and standard deviation of the relative magnitude of γ in the active and rest regions, as well as their difference and significance, for peak levels of activation of 120, 140, 160, 180, and 200% the baseline level.

	Rest		Active			
	Mean γ/*k*_2a_	SD γ/*k*_2a_	Mean γ/*k*_2a_	SD γ/*k*_2a_	Difference	*P*-value
	120%					
IFR	0.188	0.123	0.263	0.153	0.0752	0.151 (n.s.)
IFR+HYPR	0.128	0.061	0.208	0.109	0.0796	p*< 0.05
Kernel	0.0412	0.0216	0.0885	0.0173	0.0472	p**< 0.01
	140%					
IFR	0.145	0.0751	0.159	0.00849	0.0146	0.626 (n.s.)
IFR+HYPR	0.106	0.0526	0.152	0.0963	0.0459	0.164 (n.s.)
Kernel	0.0503	0.0388	0.142	0.00927	0.0919	p****< 0.0001
	160%					
IFR	0.155	0.0672	0.223	0.0369	0.0674	p**< 0.01
IFR+HYPR	0.0912	0.0397	0.183	0.0252	0.0923	p****< 0.0001
Kernel	0.0333	0.0109	0.199	0.0134	0.166	p****< 0.0001
	180%					
IFR	0.175	0.0852	0.282	0.0471	0.107	p** < 0.01
IFR+HYPR	0.0950	0.0606	0.238	0.0360	0.143	p****< 0.0001
Kernel	0.0370	0.0267	0.250	0.0180	0.213	p****< 0.0001
	200%					
IFR	0.145	0.0747	0.330	0.0420	0.185	p****< 0.0001
IFR+HYPR	0.0954	0.0501	0.271	0.0284	0.176	p****< 0.0001
Kernel	0.0451	0.0231	0.295	0.0136	0.250	p****< 0.0001

*Calculated from simulations with 40 million counts and considering 10 realizations per activation level. Reconstructed with independent frame reconstruction (IFR), independent frame reconstruction with HYPR denoising (IFR+HYPR), and spatiotemporal kernel reconstructions (Kernel). n.s: not significant.*

### Awake Rat Cold Raclopride Brain Scans

**FIGURE 6:**
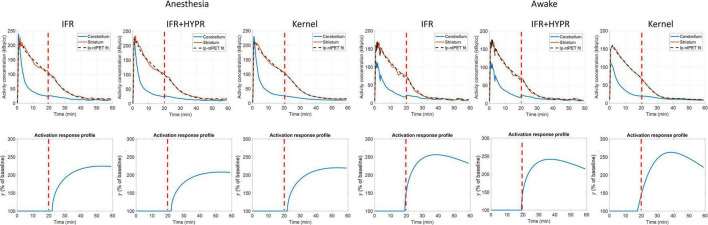
Regional striatum and cerebellum TACs calculated from independent frame reconstruction (IFR), independent frame reconstruction with HYPR denoising (IFR+HYPR), and spatiotemporal kernel reconstructions (Kernel), in cold raclopride challenge scans of anesthetized and awake rats. Activation response profiles calculated from the lp-ntPET modeling are shown below. Cold raclopride injection at 20 min (red dotted line).

Figure 6 shows the regional striatum and cerebellum TACs as well as the kinetic modeling fit and ARP, in both anesthetized and awake rats using IFR, IFR+HYPR, and Kernel. Noise is greater in Awake IFR, compared to Anesthesia IFR TACs, with both IFR+HYPR and Kernel reducing noise in both cases. ARP calculated from IFR, IFR+HYPR, and Kernel fits have very good correspondence, but IFR+HYPR shows reduced magnitude compared to IFR and Kernel. The ARP from awake rats have a sharper profile and larger relative magnitude compared with anesthesia ARP. Table 4 shows the parameters of the lp-ntPET kinetic modeling. Anesthesia scan parameters *k*_*2a*_, and γ have good correspondence between IFR, IFR+HYPR and Kernel, but confidence intervals (95%) are smaller for values calculated using Kernel. Difference in awake scans *k*_*2a*_, and γ are larger between IFR and Kernel (12 and 15% difference, respectively), with IFR+HYPR showing intermediate values. Timing parameters *t*_*d*_, and *t*_*p*_ are the same using IFR, IFR+HYPR but slightly change using Kernel, for which confidence intervals are smaller.

**TABLE 4:** Regional striatum *k*_*2a*_, γ, *t*_*d*_, and *t*_*p*_ parameters calculated with the lp-ntPET kinetic model, using independent frame reconstruction (IFR), independent frame reconstruction with HYPR denoising (IFR+HYPR), and spatiotemporal kernel reconstructions (Kernel), for the anesthetized and awake rats.

	Anesthesia			Awake		
	IFR	IFR+HYPR	Kernel	IFR	IFR+HYPR	Kernel
*k*_*2a*_(1/min)	0.135 ± 0.0392	0.150 ± 0.0406	0.132 ± 0.0266	0.124 ± 0.0928	0.118 ± 0.0863	0.109 ± 0.0270
γ (1/min)	0.174 ± 0.0787	0.164 ± 0.0759	0.159 ± 0.0508	0.215 ± 0.106	0.191 ± 0.0923	0.182 ± 0.0393
*t*_*d*_ (min)	17.5 ± 6	17.5 ± 6	19 ± 5.25	17.5 ± 6.75	17.5 ± 6.75	16 ± 6
*t*_*p*_ (min)	49 ± 6	49 ± 6	49 ± 5.25	37 ± 8.25	35.5 ± 7.5	37 ± 6.75

*Uncertainty is calculated using the ABC algorithm.*

Supplementary Figures 5, 6 show the posterior distribution of parameters estimated with ABC, in anesthesia and awake scans, respectively. In the anesthesia scan, compared to IFR, IFR+HYPR slightly decreases distribution spread, with Kernel showing distributions with the smallest spread in all parameters. In the awake scan the same trend is observed, where the peak of the distributions in timing parameters is more clear with the Kernel method.

**FIGURE 7:**
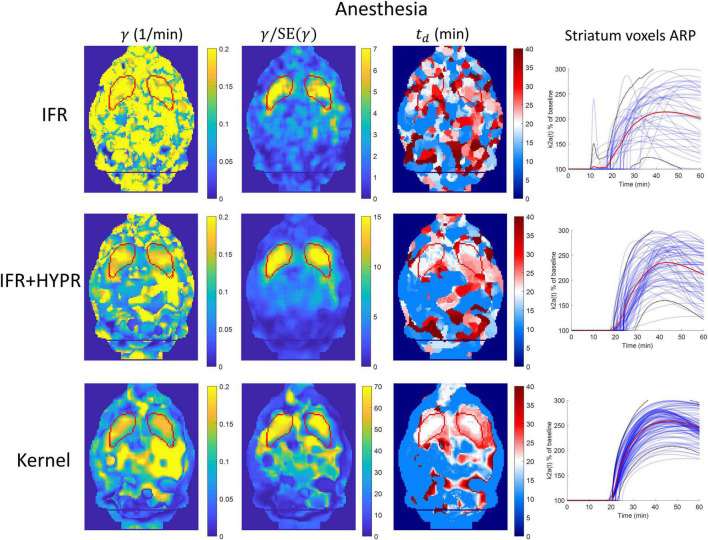
Parametric γ, γ/SE(γ) (γ t-statistic), and *t*_*d*_brain maps, as well as activation response profiles (ARP) for striatum voxels, calculated using independent frame reconstruction (IFR, first row), independent frame reconstruction with HYPR denoising (IFR+HYPR, middle row), and spatiotemporal kernel reconstruction (Kernel, third row), for the anesthetized rat scan. White regions in *t*_*d*_ maps correspond to cold raclopride injection time (20 min). Striatum delineated from MRI template shown in red.

**FIGURE 8:**
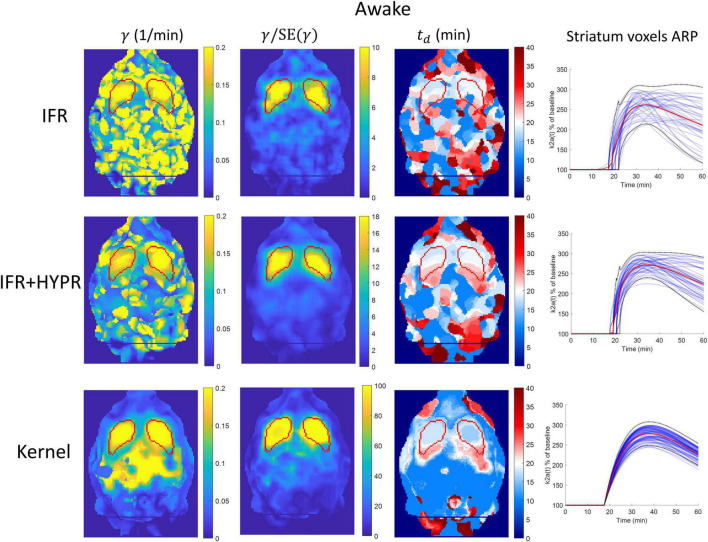
Parametric γ, γ/SE(γ) (γ t-statistic), and *t*_*d*_brain maps, as well as activation response profiles (ARP) for striatum voxels, calculated using independent frame reconstruction (IFR, first row), independent frame reconstruction with HYPR denoising (IFR+HYPR, middle row), and spatiotemporal kernel reconstruction (Kernel, third row), for the awake rat scan. White regions in *t*_*d*_ maps correspond to cold raclopride injection time (20 min). Striatum delineated from MRI template shown in red.

Figure 7 shows the γ, γ/SE(γ) (γ t-statistic), and *t*_*d*_ parametric maps for the anesthesia scan, as well as striatum voxel-wise ARP, calculated using IFR, IFR+HYPR, and Kernel reconstructions. Figure 8 shows the equivalent figure for the awake scan. The shape of the striatum is not visible from the IFR anesthesia γ parametric map, showing high intensity values throughout the brain, while HYPR denoising slightly improves striatum structure. With Kernel the striatum structure is visible in the γ map, but also showing high intensity values outside the striatum. The IFR γ t-statistic parameter map shows lower intensity values outside the striatum, and some regions of high intensity within the striatum. HYPR denoising further improves striatum structure and reduces noise outside striatum. The same effect is observed in the Kernel γ t-statistic map, but showing a better striatum structure shape and slightly higher intensity values outside the striatum. The onset time *t*_*d*_ parametric map shows no striatum structure at the cold raclopride injection time value (20min, assigned to white color) using IFR and IFR+HYPR, while *t*_*d*_ maps using Kernel show good correspondence at the striatum region with the cold raclopride injection time. This is also shown in the ARP curves calculated from striatum voxels TACs, with IFR showing large variation across voxels, which is reduced using IFR+HYPR, and further reduced using Kernel. Supplementary Figure 7 shows histograms of γ, *t*_*d*_, and *t*_*p*_ values considering striatum voxels. Distribution of values is larger in IFR histograms, especially in the *t*_*d*_ histogram where no clear distribution centered at a single value can be discerned. HYPR denoising reduces spread, and Kernel histograms show distributions with the lowest spread, with clear peaks in γ and *t*_*d*_ histograms (0.16 min^–1^, and 20 min, respectively), close to the regional analysis values (0.16 min^–1^, and 22 min).

As in the anesthesia scan, in awake γ parametric maps using IFR the striatum structure cannot be identified, with HYPR denoising reducing noise outside the striatum. Using Kernel, the striatum structure is better defined, but also showing high values outside the structure. Calculating the γ t-statistic map reduces high intensity values outside the striatum for all methods, and the striatum structure is visible using IFR and IFR+HYPR, but shape is better defined using Kernel. The IFR and IFR+HYPR *t*_*d*_ parametric map show regions within the striatum with values close to the true cold raclopride injection time (white regions), but striatum shape is not well defined. On the other hand, Kernel *t*_*d*_ map white regions are well defined within the striatum. Voxel-wise striatum ARP variation is reduced using Kernel compared with IFR and IFR+HYPR. Supplementary Figure 8 shows γ, *t*_*d*_, and *t*_*p*_ histograms considering striatum voxels. Histogram distributions are improved with HYPR denoising, but have less spread using Kernel, particularly in γ and *t*_*p*_ histograms. Peak value of *t*_*p*_ in the IFR and IFR+HYPR histograms (30 min) shows a large difference with respect to the region analysis value (38.5 min), while Kernel *t*_*p*_ histogram peak value has a closer value (38 min).

## Discussion

The spatiotemporal kernel method has been validated for lp-ntPET kinetic modeling and adapted in conjunction with motion correction reconstruction, reducing noise in dynamic reconstructions and in kinetic parameters. Depending on the level of noise, subtle transient changes in neurotransmitter levels might be difficult to detect using regular independent frame reconstruction. The spatiotemporal kernel reconstruction reduces noise in the dynamic PET data, therefore allowing one to detect changes in the time activity curves and calculate kinetic parameters with less uncertainty. This was validated in simulation experiments, and then applied in a real data awake rat experiment.

For simulations data, at the pixel level Kernel produce reconstructions TACs with similar noise to IFR TACs at the regional level. Some negative bias is present in γ, at the regional and pixel level using IFR+HYPR and Kernel, which can be observed in the ARP lower magnitude compared to noise-free ARP (Figure 4). This could be caused by some smoothing effect of the HYPR filtering and Kernel reconstruction in the TAC. For HYPR this could be caused by the mismatch between the composite image and the lower intensity voxels in the release frames, while for Kernel the contrast can be adjusted by fine-tuning the spatial kernel matrix threshold (Wang and Qi, 2015). However, timing parameters (*t*_*d*_ and *t*_*p*_) have excellent statistics (low coefficient of variation and bias) in both regional and pixel-wise data using Kernel. Although variability in the TAC magnitude increases with noise level using Kernel, the overall shape is well preserved, which might be an important factor in calculating accurate lp-ntPET kinetic modeling timing parameters. On the other hand, noise in IFR TACs can be overfitted using lp-ntPET and wrongly interpreted as an activation by the model. This is shown in the high variability in the IFR ARP parameters at the pixel level.

Similarly, parametric γ, *t*_*d*_ and *t*_*p*_ maps present lower noise using Kernel compared with IFR and IFR+HYPR, and have less variation across noise levels. But HYPR denoising presents closer performance to Kernel. At reduced count levels magnitude of γ parametric maps increase with respect to high count levels, but this effect is less pronounced using Kernel compared with IFR and IFR+HYPR. Activation onset time *t*_*d*_ parametric maps calculated using IFR present larger bias with increasing noise, but have stable values across different noise levels using Kernel. For peak time *t*_*p*_ parametric maps, mean values are similar between IFR, IFR+HYPR and Kernel maps, but noise is lower using IFR+HYPR and Kernel. As also observed in the pixel-wise analysis, this indicates that peak time *t*_*p*_ might be the most robust parameter calculated with lp-ntPET kinetic modeling.

The spatiotemporal kernel reconstruction also allows to detect more subtle activation profiles, as observed in the simulations with different ARP magnitudes. At the lowest activation of 120% the baseline level, kinetic modeling with lp-ntPET using IFR+HYPR and Kernel produced relative activation magnitudes (γ/*k*_2*a*_) in the active region significantly different from the rest region, but IFR+HYPR failed to show significant differences at 140% the baseline level. The significance and magnitude of the difference increased with largest activation peak value using Kernel. Using IFR on the other hand, only for activations of 160% baseline and larger, a significant difference in the relative activation magnitude was observed between rest and active regions. Reduced noise in the Kernel reconstruction would therefore allow to detect neurotransmitter release of less intensity compared with IFR.

In anesthesia and awake cold raclopride scans, there is good correspondence between IFR, IFR+HYPR and Kernel lp-ntPET parameters at the regional level, with less noisy TACs calculated using Kernel. *k*_*2a*_ and γ parameters confidence intervals calculated using ABC (Fan et al., 2021) are smaller using Kernel compared with IFR and IFR+HYPR due to the TACs lower noise. For all methods, the activation due to the cold raclopride challenge has larger relative magnitude and faster rise in the awake compared to the anesthesia scan. This can also be observed in the striatum TACs where the activity level reaches the cerebellum level at an earlier time in the awake scan compared to the anesthesia scan. Due to the relatively large volume of the rat striatum structure (0.043 cm^3^ single side), regional analysis performs well using IFR. However, for smaller structures, or for studies with lower activity injection, the spatiotemporal kernel method would show larger differences compared with independent frame reconstruction (Miranda et al., 2021).

At the voxel level, performance of IFR is suboptimal, with HYPR denoising improving performance. The striatum structure cannot be identified in the IFR and IFR+HYPR activation magnitude γ parametric maps, but it is visible in the Kernel γ parametric maps, although large intensity values are still present outside the striatum, in both anesthesia and awake scans. Calculating the γ t-statistic helps to better identify the striatum structure in parametric maps using all methods, reducing high intensity regions outside the striatum, but striatum shape is better defined using Kernel. Activation onset time *t*_*d*_ parametric maps also show no striatum structure using IFR and IFR+HYPR, but it is well identified using Kernel by looking at the voxels with values close to the true cold raclopride injection time (20 min). Looking at the striatum voxels ARP, variation is large across voxels using IFR, and reduced with HYPR denoising, but more consistent ARP are obtained using Kernel, and consequently more consistent kinetic parameters are obtained. This is also shown in the γ, *t*_*d*_, and *t*_*p*_ histogram plots with less spread values using Kernel compared with IFR and IFR+HYPR.

The spatiotemporal kernel reconstruction benefits from the relatively large rat striatum size spanning several voxels (about 100), which allows calculation of spatial basis functions also spanning several voxels. In addition, the temporal basis functions spanning only a finite time interval allow to preserve the shape of the TAC, including the transient changes. Temporal noise in the IFR voxel-wise TACs is detrimental for the lp-ntPET kinetic modeling since noise can be overfitted and wrongly interpreted as an activation in the TAC. Additional incorporation of HYPR denoising in calculation of the kernel matrix could further improve performance of the Kernel method (Cheng et al., 2021; Miranda et al., 2021).

Future work involves using the spatiotemporal kernel reconstruction in studies that could present subtle activations, for example in drug challenge (Kyme et al., 2019) or behavioral studies (Koepp et al., 1998; Lataster et al., 2011).

## Conclusion

The spatiotemporal kernel reconstruction (Kernel) has been validated for lp-ntPET kinetic modeling and adapted for PET brain motion correction reconstruction in freely moving animals. In simulation experiments, Kernel improves kinetic parameters noise and bias, and allows to detect neurotransmitter activations of lower magnitude, compare with independent frame reconstruction (IFR) and HYPR denoising. In anesthetized and awake rat experiments, Kernel produce lp-ntPET parametric maps with better definition of the striatum structure and with more consistent parameter values across striatum voxels compared with IFR. Noise reduction using Kernel allows to perform neurotransmitter activation studies with lower parameters noise, and with detection of subtle neurotransmitter activations.

## Data Availability Statement

The raw data supporting the conclusions of this article will be made available by the authors, without undue reservation.

## Ethics Statement

The animal study was reviewed and approved by Animal Experimental Ethical Committee of the University of Antwerp.

## Author Contributions

AM was involved on the experimental design, software writing, data analysis and manuscript drafting and editing. JV was involved in the experimental design, data analysis, and manuscript drafting. DB, SiS, and StS were involved in drafting and editing the manuscript and figures. All authors approved the final manuscript and they are accountable for the content of the work.

## Conflict of Interest

The authors declare that the research was conducted in the absence of any commercial or financial relationships that could be construed as a potential conflict of interest.

## Publisher’s Note

All claims expressed in this article are solely those of the authors and do not necessarily represent those of their affiliated organizations, or those of the publisher, the editors and the reviewers. Any product that may be evaluated in this article, or claim that may be made by its manufacturer, is not guaranteed or endorsed by the publisher.
