# Supplementary material for: Spatiotemporal Kernel Reconstruction for Linear Parametric Neurotransmitter PET Kinetic Modeling in Motion Correction Brain PET of Awake Rats
Source: Front Neurosci. 2022 May 12;16:901091. doi: 10.3389/fnins.2022.901091 (PMC9133502; doi:10.3389/fnins.2022.901091)
Supplement: Supplementary file 1 [file Data_Sheet_1.PDF]

Supplementary Material

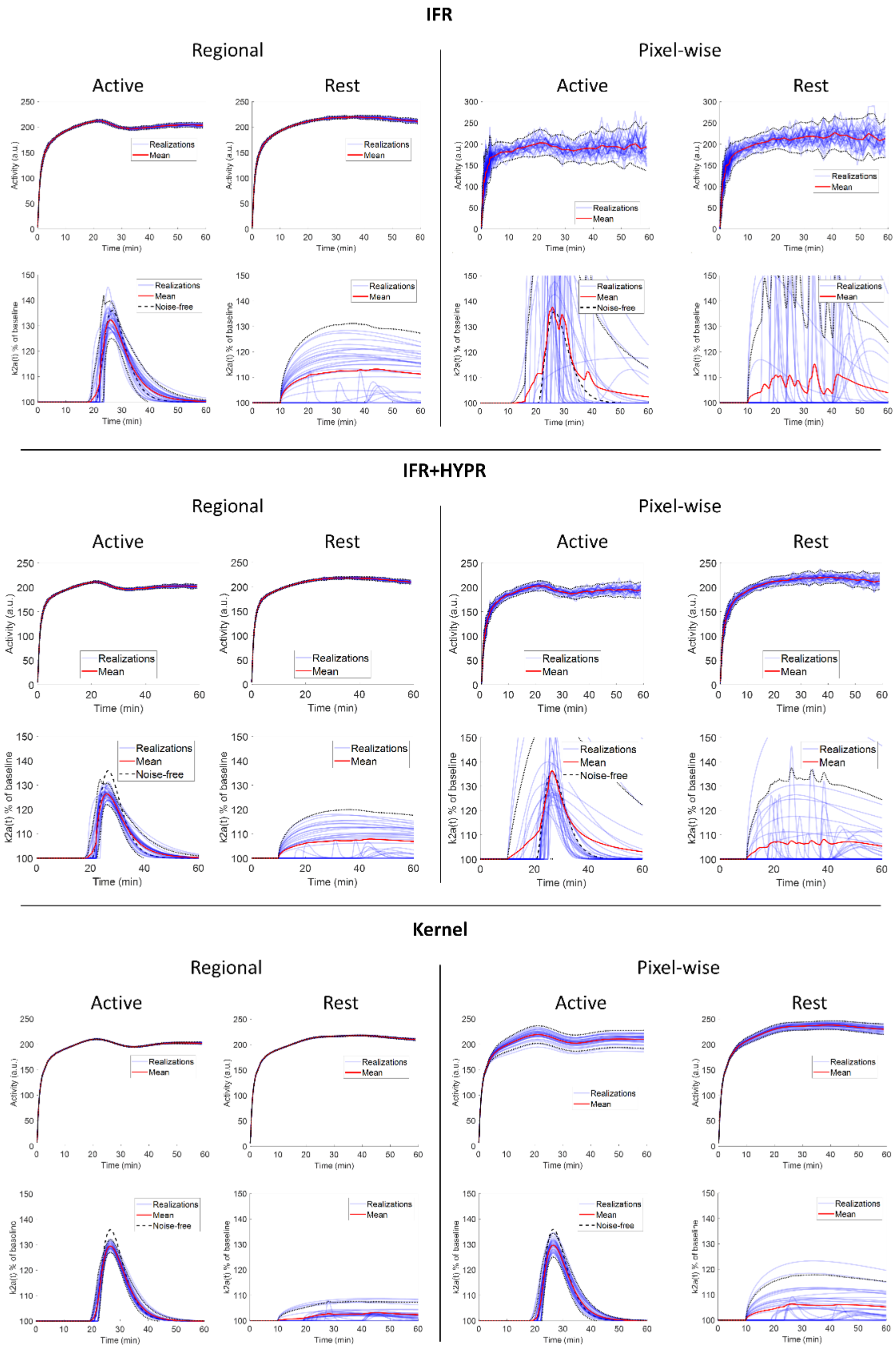

Supplementary Figure 1. Individual for every realization and mean regional and voxel-wise TACs (row 1 and 3) and activation response profiles (row 2 and 4), for the active and rest regions, using independent frame reconstruction (IFR), independent frame reconstruction with HYPR denoising (IFR+HYPR), and spatiotemporal kernel reconstruction (Kernel), for simulations with 40 million counts. Dotted lines show ±2 std.

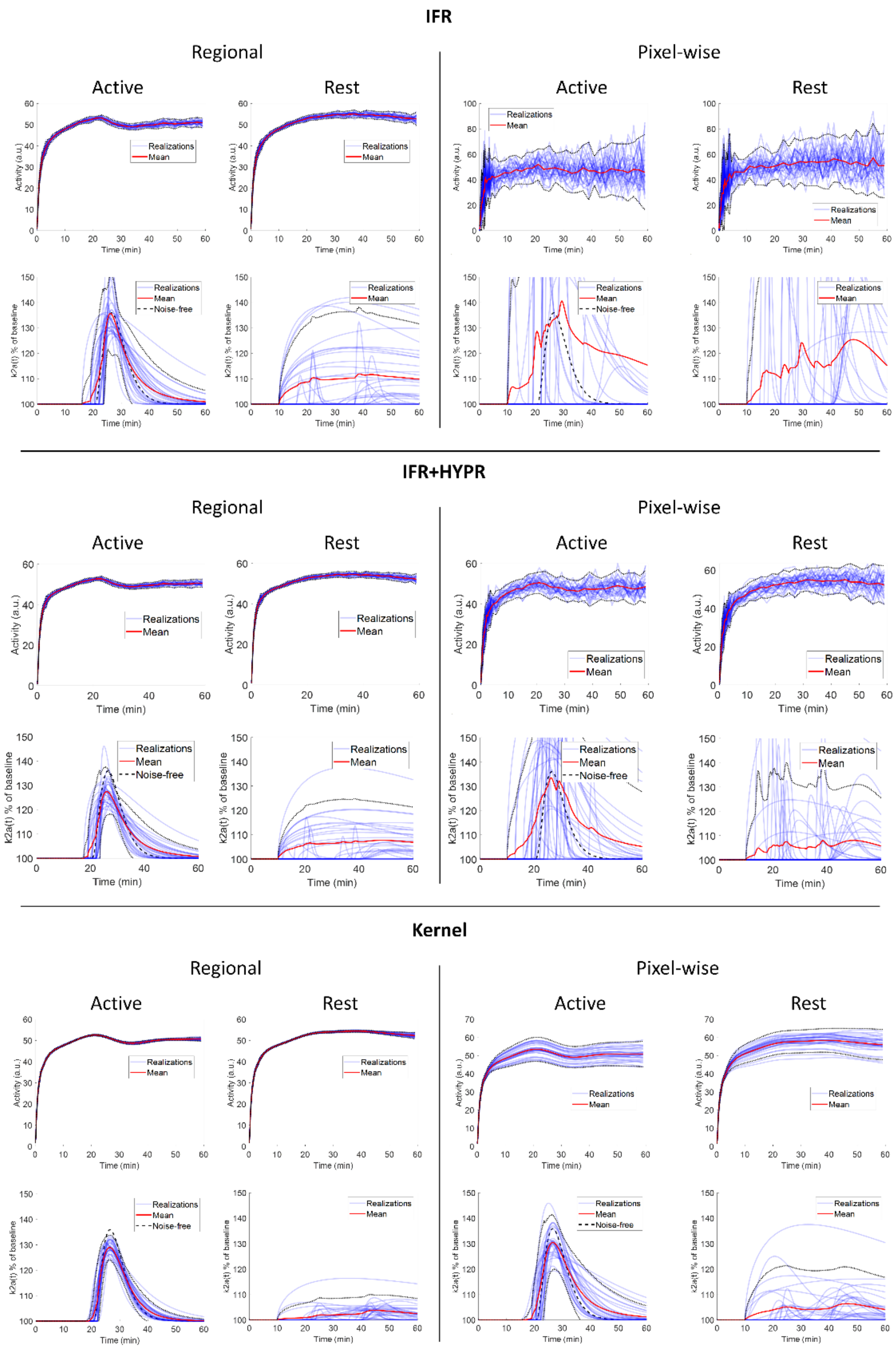

Supplementary Figure 2. Individual for every realization and mean regional and voxel-wise TACs (row 1 and 3) and activation response profiles (row 2 and 4), for the active and rest regions, using independent frame reconstruction (IFR), independent frame reconstruction with HYPR denoising (IFR+HYPR), and spatiotemporal kernel reconstruction (Kernel), for simulations with 10 million counts. Dotted lines show ±2 std.

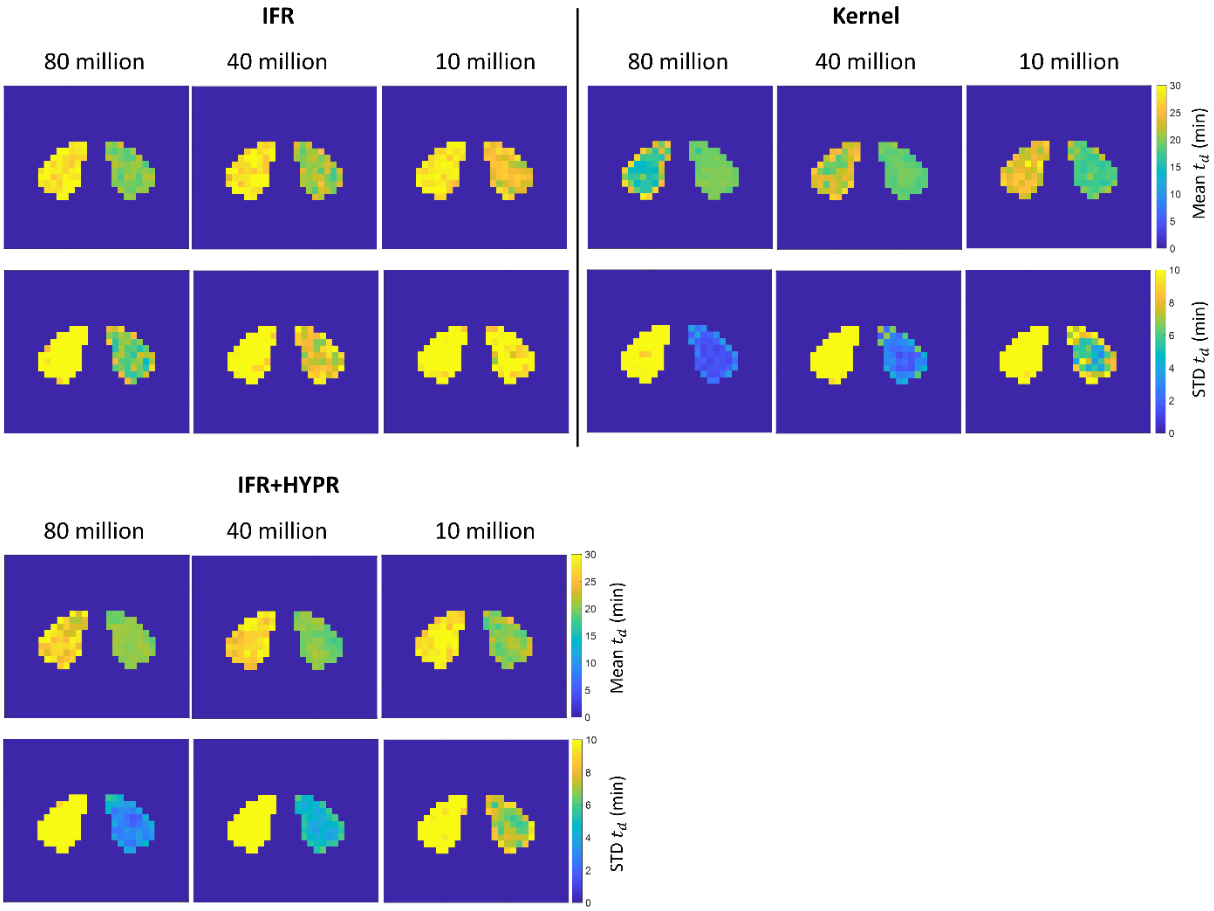

Supplementary Figure 3. Rest and active parametric t_d mean (top row) and standard deviation (bottom row) images, for independent frame reconstruction (IFR), for independent frame reconstruction with HYPR denoising (IFR+HYPR), and spatiotemporal kernel reconstructions (Kernel).

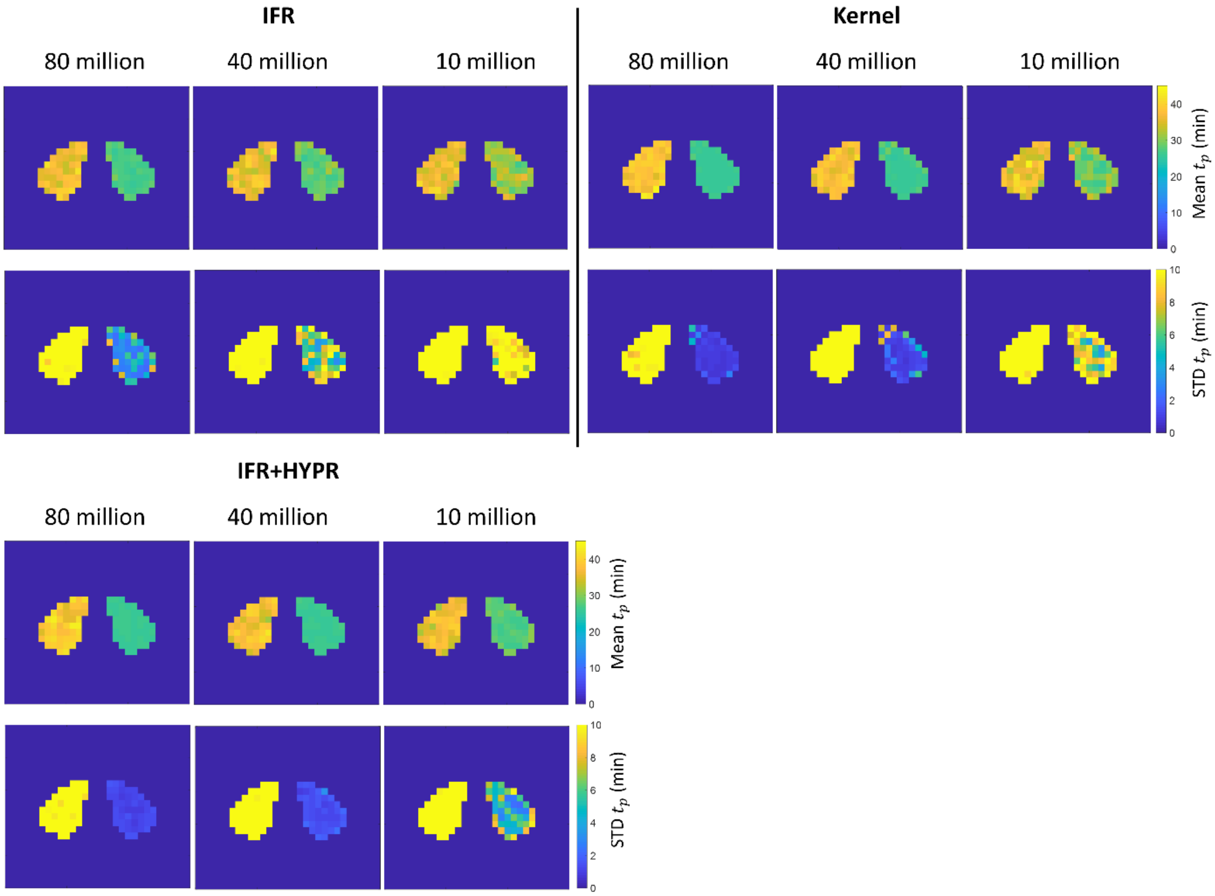

Supplementary Figure 4. Rest and active parametric t_p mean (top row) and standard deviation (bottom row) images, for independent frame reconstruction (IFR), for independent frame reconstruction with HYPR denoising (IFR+HYPR), and spatiotemporal kernel reconstructions (Kernel).

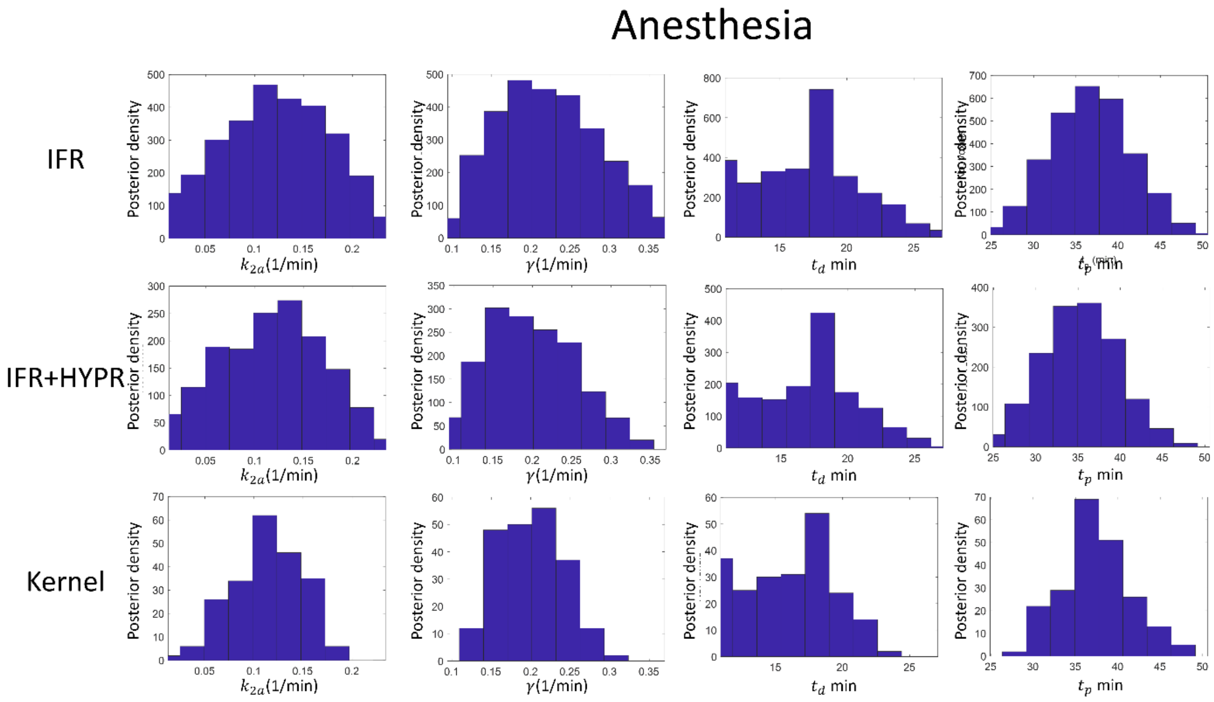

Supplementary Figure 5. Posterior distribution of k_{2a}, γ, t_d, and t_p parameters calculated with the ABC algorithm, in IFR, IFR+HYPR, and Kernel reconstruction, in the rat anesthesia scan with cold raclopride injection.

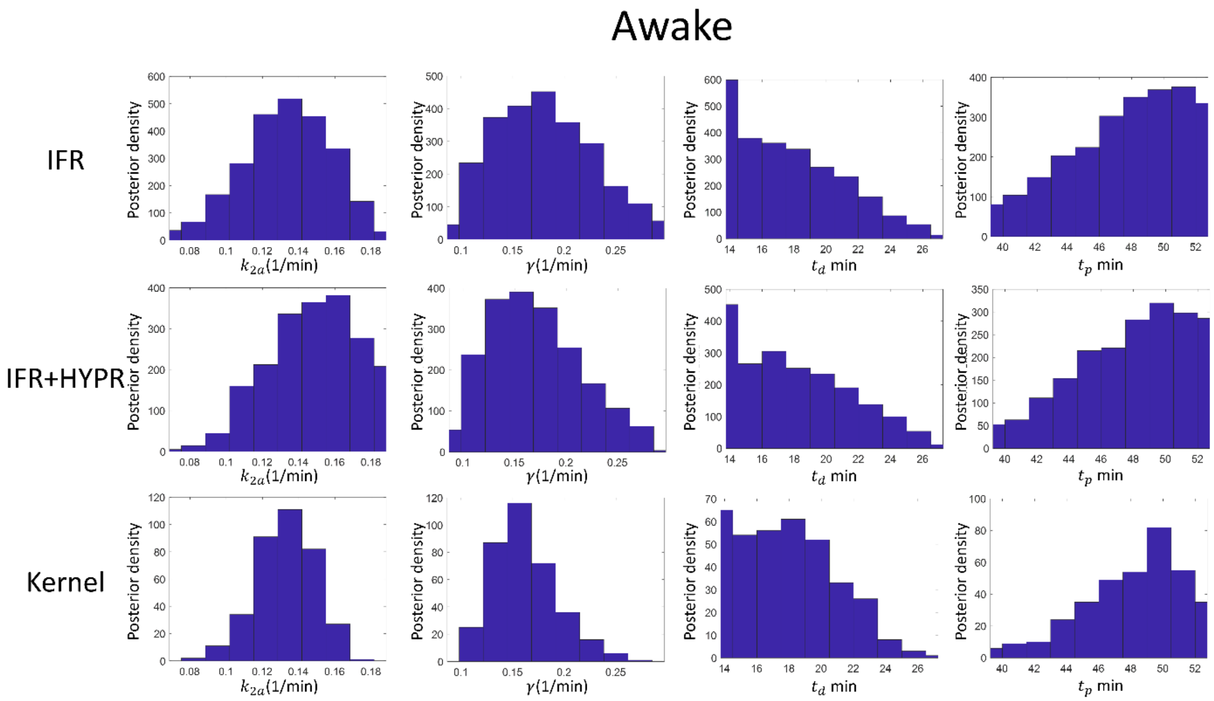

Supplementary Figure 6. Posterior distribution of k_{2a}, γ, t_d, and t_p parameters calculated with the ABC algorithm, in IFR, IFR+HYPR, and Kernel reconstruction, in the rat awake scan with cold raclopride injection.

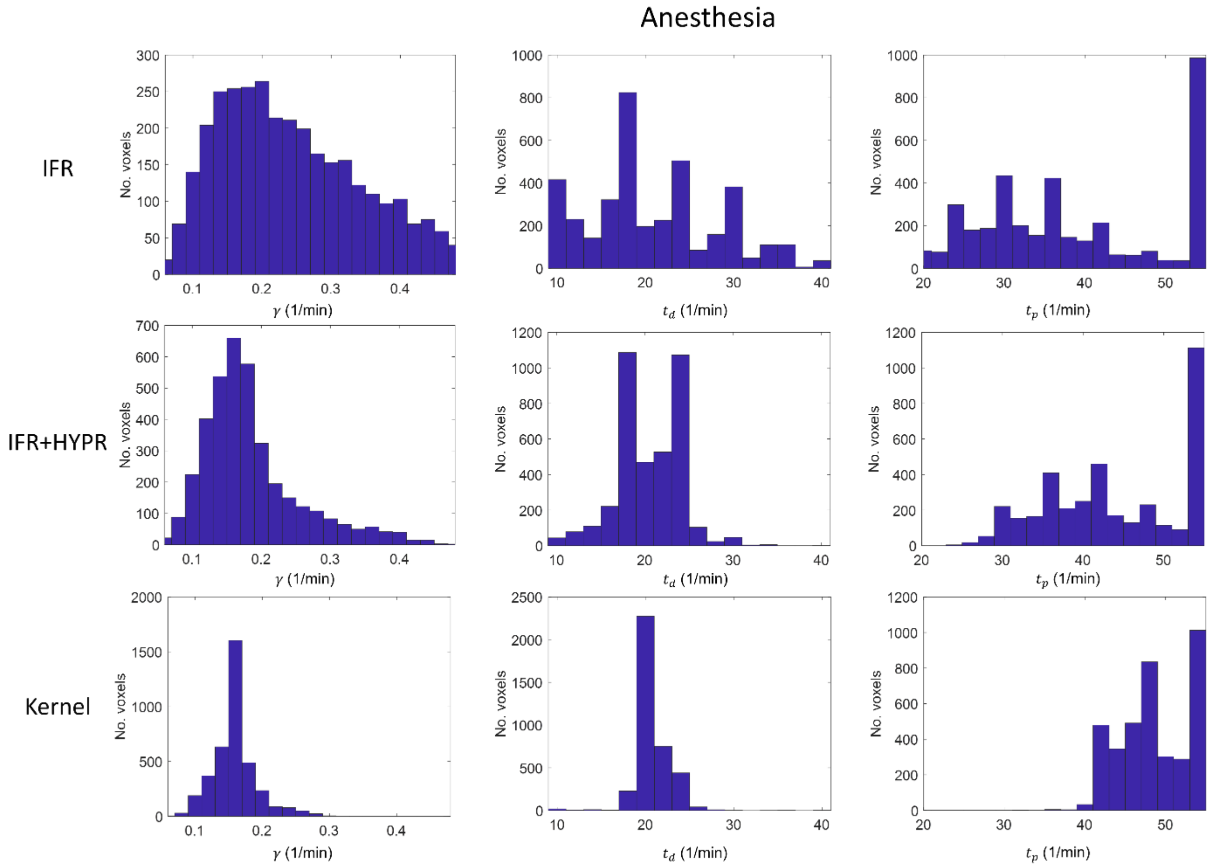

Supplementary Figure 7. Histogram of γ, t_d, and t_p values considering striatum voxels from the anesthesia scan, using independent frame reconstruction (first row, IFR), for independent frame reconstruction with HYPR denoising (middle row, IFR+HYPR), and the spatiotemporal kernel reconstruction (third row, Kernel).

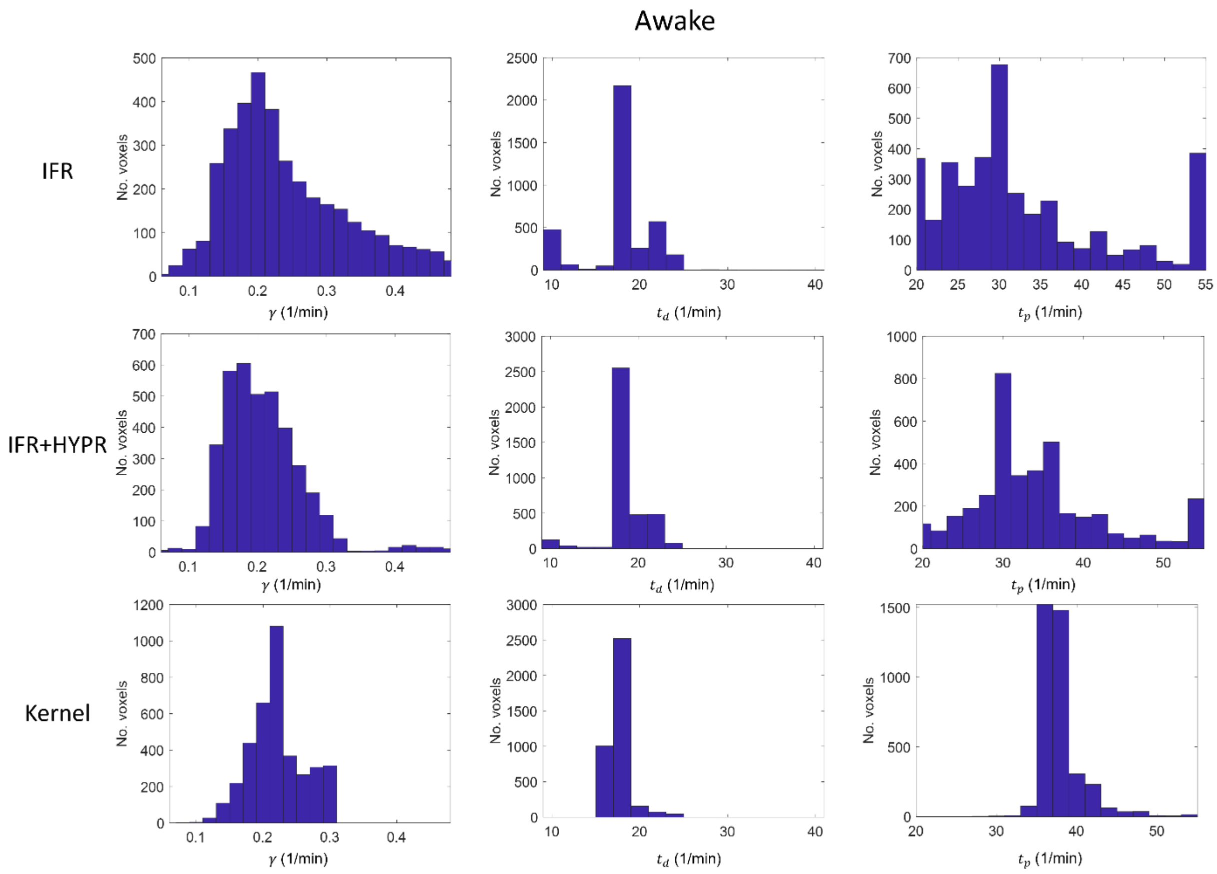

Supplementary Figure 8. Histogram of γ, t_d, and t_p values considering striatum voxels from the awake scan, using independent frame reconstruction (first row, IFR), for independent frame reconstruction with HYPR denoising (middle row, IFR+HYPR), and the spatiotemporal kernel reconstruction (third row, Kernel).
